# Management of Intraductal Papillary Mucinous Neoplasms: Controversies in Guidelines and Future Perspectives

**DOI:** 10.1007/s11938-018-0190-2

**Published:** 2018-09-08

**Authors:** IJM Levink, MJ Bruno, DL Cahen

**Affiliations:** Department of Gastroenterology and Hepatology, Erasmus University Medical Centre, Floor Na-6, Doctor Molewaterplein 40, 3015 GD Rotterdam, the Netherlands

**Keywords:** Pancreatic cyst, Intraductal papillary mucinous neoplasm, IPMN, Management, Guideline, Diagnosis

## Abstract

**Purpose of review:**

Management of intraductal papillary mucinous neoplasm (IPMN) is currently based on consensus, in the absence of evidence-based guidelines. In recent years, several consensus guidelines have been published, with distinct management strategies. In this review, we will discuss these discrepancies, in order to guide treating physicians in clinical management.

**Recent findings:**

The detection rate of pancreatic cysts has increased substantially with the expanded use of high-quality imaging techniques to up to 45%. Of these cysts, 24–82% are IPMNs, which harbour a malignant potential. Timely detection of high-risk lesions is therefore of great importance. Surgical management is based on the presence of clinical and morphological high-risk features, yet the majority of resected specimens appear to be low risk.

**Summary:**

International collaboration and incentive large-scale prospective registries of individuals undergoing cyst surveillance are needed to accumulate unbiased data and develop evidence-based guidelines. Additionally, development of non-invasive, accurate diagnostic tools (e.g. biomarkers) is needed to differentiate between neoplastic and non-neoplastic pancreatic cysts and detect malignant transformation at an early stage (i.e. high-grade dysplasia).

## Introduction

Intraductal papillary mucinous neoplasm (IPMN) is a pancreatic cystic lesion originating from intraductal growth of mucin producing cells. In 1980, Ohhashi et al. [1] were the first to describe IPMN. In 1996, it was recognised as a separate entity [2, 3]. The increased detection and awareness of IPMNs led to the development of several, mainly consensus-based, periodically revised national and international guidelines [4•, 5•, 6•, 7•, 8•, 9•, 10•]. Notably, evidence is mainly based on surgical cohorts and information on patients managed conservatively is limited.

## Classification

Based on localization and extent, three subtypes can be identified; main-duct (MD-IPMN), branch-duct (BD-IPMN) and mixed-type IPMN (MT-IPMN). Every subtype exhibits a certain risk of malignancy and requires a specific therapeutic approach.

MD-IPMN is recognised as dilation (segmental or diffuse) of the main pancreatic duct (MPD) of > 5 mm, for which other causes of ductal obstruction have been ruled out, is mostly located in the pancreatic head (64–67%) and accounts for 15-21% of the IPMNs [11–13]. It has the highest risk to exhibit malignant disease (28–81%) [10•, 12–20]. Therefore, an MPD diameter ≥ 10 mm is considered an absolute indication for surgical resection [10•, 21]. Approximately 70% of patients is symptomatic [22].

BD-IPMN is defined as a grape-like cyst (> 5 mm) that communicates with the MPD [12, 13]. It accounts for 41–64% of IPMNs and can develop multifocally throughout the pancreas, with a preference for the uncinate process [11, 12]. BD-IPMNs have the least risk of malignant progression (7–42%), yet their multifocality (40%) and high post-surgical recurrence rate (7–8%) are insidious. Interestingly, it has not been proven that multifocality increases the risk of malignancy [10•, 12–20, 23, 24]. The indication for surgical resection depends on the presence of high-risk clinical and morphological features [6•, 10•].

MT-IPMN meets both criteria of MD- and BD-IPMN and is seen in 22–38% of cases, of which 20–65% are malignant [12, 13, 15, 16, 18, 19, 25–27]. The therapeutic approach is the same as for MD-IPMN [6•, 10•]. Potential overlap between these groups should be taken into account, since 29% of patients with BD-IPMN appear to have MPD involvement after resection [23].

IPMN is also classified according to its cellular morphology as gastric, intestinal, cholangio-papillary or oncocytic type. This classification is based on mucin (MUC) gene expression, architecture and cytology, yet different subtypes can be seen in the same cyst. Each type exhibits a particular risk of malignancy (Table 1).

**Table 1:** Characteristics of IPMN based on cellular morphology (data from surgical series) [28–30]

	Gastric type	Intestinal type	Pancreatobiliary type	Oncocytic type
Morphology	Thick finger-like papillae	Villous papillae	Complex thin branching papillae	Complex thick papillae with eosinophilic oncocytic cells
MUC gene expression				
- MUC 1	−	−	+	−/+
- MUC 2	−	+	−	−/+
- MUC 5AC	+	+	+	+
- MUC 6	+	−/+	−/+	−
Percentage of IPMNs	46–63%	18–36%	7–18%	1–8%
Location				
- Head	69–72%	64–67%	63–67%	25–33%
- Body or tail	28–31%	33–37%	34–37%	67–75%
Main-duct involvement	19%	63%	50%	38%
Invasive progression	10%	40%	68%	50%
Type of adenocarcinoma	Tubular (79%)	Colloid > tubular	Tubular (82%)	Tubular > colloid
Mural nodules	30%	56%	57%	100%
Recurrence rate	9%	20%	46%	14%
5-year survival	85%	85%	54%	79%

*IPMN*, intraductal papillary mucinous neoplasm

## Risk factors

Both the risk of IPMN development and malignant degeneration increase with age [12, 15, 17, 19, 20, 31]. The mean age at time of IPMN detection is 65 years. There is a small male gender predisposition [12, 19, 20]. Also, lifestyle is of influence, as smoking and alcohol abuse increase the risk of having high-risk and worrisome features [11, 31]. Increased BMI and the associated presence of abdominal fat are known to play a role in the development of other pancreatic diseases (e.g. type-2 diabetes mellitus (DM) and pancreatic ductal adenocarcinoma (PDAC)), due to fatty infiltration and inflammation [32, 33]. Yet, knowledge about the relation between abdominal fat, IPMN and subsequent malignant transformation is limited. Sturm et al. (2013) [34] found a relation between severe obesity (BMI ≥ 35) and an increased risk of malignant transformation in IPMN (OR 10.1, 95% CI 1.30–78.32) [31, 35].

There is a causative link between IPMN and DM. Of patients with IPMN, 10–45% have diabetes [11–14, 16, 19, 31, 36, 37] and in the case of diabetes, the risk of detecting IPMN is higher (OR 1.79; 95% CI 1.08–2.98) [35], especially in the case of insulin-use (OR 6.03, 95% CI 1.74–20.84) [35]. In reverse, the presence of DM is associated with a higher risk of HGD (OR 2.02, 95% CI 1.02–4.01) and carcinoma (OR 2.05, 95% CI 1.08–3.87) [38]. Additionally, patients with chronic pancreatitis have an increased risk of IPMN (OR 10.1, 95% CI 1.30–78.32) [31, 35].

Furthermore, having a family history of PDAC or another hereditary risk may pose a threat. Capurso et al. (2013) [35] compared 390 patients with IPMN with matched controls and found that 5.5% of the patients with IPMN and just 1.6% of the healthy controls had a 1st degree family member with PDAC (OR 2.94 95% CI 1.17–7.39 p 0.022) [31]. It is unknown whether patients with a positive family history have a more rapid progression. Currently, the management (surveillance and treatment), advised by clinical guidelines, is the same as for patients with sporadic IPMN [10•]. The Fukuoka guideline, however, recommends surveillance at 6-months’ intervals in patients with a positive family history with operated IPMN [6•].

## Diagnosis

### Symptoms

Most patients with IPMN are asymptomatic. Symptoms are associated with more advanced and invasive disease. Jaundice and abdominal pain are associated with invasive disease in 80 and 77% of IPMN cases, respectively. Of patients with IPMN, 13–32% are reported to present with secondary acute (recurrent) pancreatitis, although this incidence is based on surgical series and likely to be overestimated. Other symptoms are weight loss, new-onset diabetes, steatorrhea and back pain [11–15, 17–20, 31, 37, 39–41].

### Imaging techniques

**Fig. 1:**
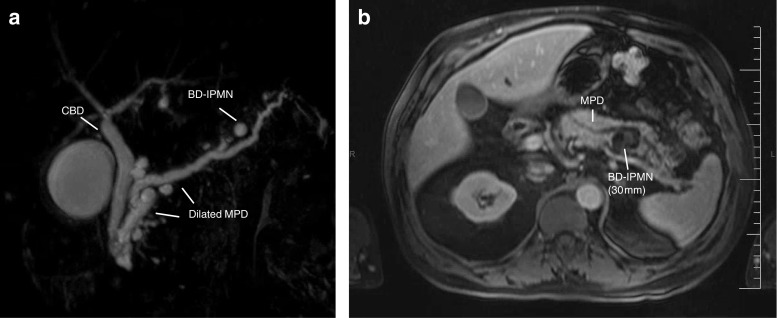
**a** MRCP—diluted pancreatic duct and Santorini with distal a diameter of less than 1 cm. Also, the image of multifocal small sidebranch IPMN. **b** MRI—ductus pancreaticus which is irregular at the level of the corpus and tail and is slightly dilated. Multiple cysteine deviations starting from the side duct. Largest cystic lesion located in the corpus with a staining solid component. Image matching a mixed-type IPMN with solid component as sign of a possible malignant degeneracy. PA—after pancreatic tail and spleen resection: the ductus pancreaticus and side branches show mixed-type IPMN, both gastric and pancreatobiliary type, with moderate dysplasia; there are extensive regressive changes with mucinous extravasation and fibrosis. No high-grade dysplasia, no malignancy

Currently, cross-sectional imaging plays a main role in lesion detection and differentiation. MRI (combined with MRCP) is the modality of choice, because of its superiority in cyst differentiation and identification of MPD connectivity, mural nodules, and septation. [6•, 7•, 8•, 10•], as well as cyst differentiation [42] (Fig. 1). Additionally, the repetitive nature of cyst follow-up mandates a non-invasive modality to eliminate radiation exposure [6•, 10•]. However, for identification of calcifications, tumour staging or surveillance of PDAC recurrence, addition of CT is recommended by some [10•]. Secretin injection during MRCP increases the likelihood of visualising MPD communication, yet only by 5%. More studies are needed to determine whether the addition of secretin outweighs costs and prolongation of scanning time [43].

**Fig. 2:**
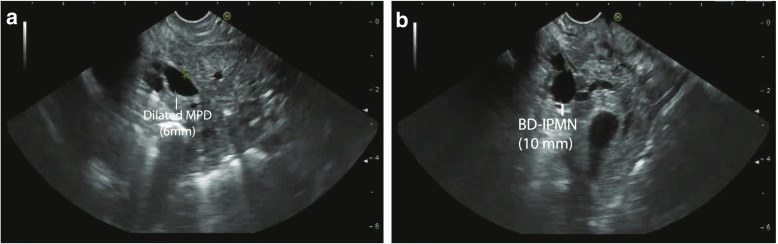
**a** and **b** EUS screenshot captured from D2, the PD is continued from the papilla: focalized dilation over a short trajectory with a diameter of 6 mm, slendering distally with a diameter of 2.7 mm. There is a homogeneous 10-mm cystic lesion not far from the papilla with a connection to de PD. No murine nodule or wall thickening. Conclusion: mixed-type IPMN in pancreatic head and uncinate process

Endoscopic ultrasound (EUS) is a good alternative for imaging. It is mainly used to assess the presence of worrisome features and should not be performed in case of an established diagnosis or clear indication for surgery. Despite a low accuracy for differentiation between cyst types (61–72%) [44, 45], it is highly appropriate for the recognition and delineation of malignant characteristics, especially intracystic structures [46–48]. Addition of contrast increases the accuracy of mural nodule detection to 98% [44] (Fig. 2).

An added benefit of EUS is that it allows for cyst fluid collection with fine-needle aspiration (FNA), which is indicated in case of indefinite imaging findings [6•, 7•, 10•]. The AGA recommends EUS-FNA in patients with a cyst diameter ≥ 3 cm, solid component or dilated MPD [8•]. The Fukuoka guideline discourages FNA in case of either high-risk or worrisome features, out of fear for tumour spill [6•]. Cytological cyst fluid analysis has a high specificity (91%), yet low sensitivity (65%) for differentiation between benign and malignant IPMN [45, 49–51]. Sensitivity may be increased if the cyst wall and solid components are also sampled [54]. The risk of complications related to cyst EUS-FNA is low (0–2.5%), although higher than for solid lesions. Potential complications are abdominal pain, bacteraemia/infection, haemorrhage and pancreatitis. Prophylactic antibiotics are recommended [51, 55–59]

### Cyst fluid analysis and biomarkers

A broad spectrum of tumour-specific (e.g. mutated KRAS and P53) and tumour-associated (e.g. CA 19-9) markers have the potential to distinguish high- from low-risk lesions and guide decision-making (Table 2) [10•]. A perfect biomarker should be detectable in an early stage and specific for pancreas neoplasia. Apart from cyst fluid, other potential biomarker sources are serum and pancreatic juice.

**Table 2:** Features suggestive for cyst-type and invasiveness [49, 58–67]

Characteristic	Pseudocyst	SCA	MCN	IPMN	Malignant IPMN
Age	> 40 years	> 60 years	Young (~ 40–50 years)	> 65 years	> 65 years
Gender	F<M	F>M	F>M (> 95%)	F~M	F~M
Symptoms	Regularly	Rare	Rare	Rare	Sometimes
Relation to acutepancreatitis	Mostly	No	No	Sometimes	Sometimes
Relation to chronic pancreatitis	Mostly	No	No	No	No
Calcifications	No	Sometimes (central)	Sometimes (peripheral)	No	No
Location	Not specific	Mostly distal	Mostly distal	Mostly proximal	Mostly proximal
Connected to MPD	No	No	No	Yes	Yes
Multifocality	No	Rare	No	Sometimes	Sometimes
Serum					
Elevated CA19-9 (> 37 U/mL)	−	−	+/−	+/−	++
Mutated KRAS	−	−	−	−	++
Mutated GNAS	−	−	−	+	+/−
Cyst fluid					
Mucin	−−	−−	++	++	++
Amylase (*>* 250 U/mL)	++	−−	−	+/−	+/−
CEA	−−	−−	+	+	++
Mutated KRAS	−−	−−	+	+	++
Mutated GNAS	−−	−−	−	++	+
Pancreatic juice					
CA19-9	−−	−−	−	+/−	+
CEA	−−	−−	−	+/−	+
Mutated KRAS	−−	−−	−	+/−	+
Mutated GNAS	−−	−−	−−	++	+/−
SMAD-4/P53	−−	−−	−−	+/−	++

*CA 19-9*, cancer antigen 19-9; *CEA*, carcino-embryonal antigen; *MPD*, main pancreatic duct; *SCA*, serous cyst adenoma; *MCN*, mucinous cystic neoplasm; *IPMN*, intraductal papillary mucinous neoplasm; *F*, female; *M*, male

Glycoproteins are often used as tumour markers. An increased serum level of CA19-9 (> 37 U/ml) is found in 85% of the patients with PDAC and is used to follow the disease course [68]. For IPMN, it is an independent predictor of malignant transformation, with a (pooled) sensitivity and specificity of 40 and 89%, respectively [69, 70]. An increased serum CA19-9 level is a relative indication for surgery and supplementary diagnostics are recommended [10]. Cyst fluid CA19-9 levels have limited clinical value for the identification of advanced neoplastic disease, yet low CA19-9 levels (< 37 U/ml) are suggestive for a non-mucinous origin [51]. Cyst fluid CEA is mainly used for cyst differentiation. A level of < 5 mg/mL is highly specific (95%) for a non-mucinous cyst and a value > 800 ng/mL for a mucinous cyst (95%) [49]. Little is known about glycoprotein detection in pancreatic juice. Hirono et al. (2012) [58] found a high accuracy (92%) for differentiation between benign and malignant IPMN, based on CEA levels in pancreatic juice (cut-off value > 30 ng/mL) [58].

Mutated genes are released after cell death and have high potential to serve as biomarkers. Tissue GNAS mutations are associated with IPMN (58–79%; OR 30, 95% CI 7.143–127.622), IPMN-associated adenocarcinoma (36%) and mucinous carcinoma (78%) [71–74]. In contrast, it is rarely detected in PDAC, PanIn-lesions and MCNs. The prevalence of GNAS mutations differs per morphological subtype: 100% in the intestinal type, 71% in the pancreatobiliary type, 51% in the gastric type and 0% in the oncocytic-type IPMN [75].

KRAS is the driver mutation in most pancreatic PDACs and is also detected in IPMN tissue (50%; OR 7.4, 95% CI 3.9–14.4) [74, 76]. However, it is less specific than GNAS, since KRAS is found in 69% of IPMN, 21% of MCN, 90% of PanIn-1 and 90% of PDAC patients [74]. The presence of tissue KRAS and GNAS gene mutations is not related to IPMN location (BD-IPMN vs. MD-IPMN) [74]. In serum, Berger et al. (2016) [77] found that total circulating cell-free DNA levels of > 0.208 ng/uL distinguish between IPMN and healthy controls with 81% sensitivity and 84% specificity, and between PDAC and healthy controls with 83% sensitivity and 92% specificity. More specifically for GNAS and KRAS, 71% of patients with IPMN harboured cell-free circulating mutated GNAS. Mutated KRAS was not detected in patients with IPMN, although it is present in 42% of patients with PDAC [77]. Adding molecular testing to clinical features and morphology increases sensitivity of IPMN and MCN differentiation to 90 and 94%, respectively. However, more research is needed to distinguish whether the clinical value outweighs the high costs of these sensitive laboratory techniques [59, 78]. For pancreatic juice, Suenaga et al. (2018) [60] found GNAS gene mutations in 70% of patients with IPMN. Also, TP53 and SMAD-4 levels were found to be related to dysplasia grade, and able to distinguish IPMN from PDAC with a sensitivity and specificity of 32 and 100%, respectively [60, 79]. A VHL gene mutation increases the probability of detecting a serous cyst neoplasia (SCN) [60, 79].

### Other techniques

Pancreatoscopy uses a thin scope that is introduced in the MPD during ERCP or surgery. It enables intraductal visualisation and image-guided tissue sampling. For differentiation between benign and malignant MD-IPMN, the accuracy is relatively high (88%), yet also are the rates of post-procedural pancreatitis (7%) [80]. During surgery, pancreatoscopy may be combined with intraductal frozen biopsies, to assess the extent of MPD involvement and guide resection [10•, 81].

Needle-based confocal laser endomicroscopy (nCLE) uses a small probe (0.85 mm) that is placed in a pancreatic cyst via a 19-gauge FNA needle and provides a real-time microscopic view (width 320 μm, resolution 3.5 μm). It is able to detect a pancreatic cystic neoplasm with a sensitivity of 59–80% and a specificity of 100%. However, it is currently discouraged by the EU guidelines due to high adverse event rates (7–9%) [10•, 82–85].

## Clinical strategy and surveillance

### Surveillance

Nowadays, surveillance is recommended in patients with (operated) pancreatic cysts suspected for MCN or IPMN. The best utility and manner of surveillance have not been established. At present, surveillance is based on consensus guidelines, namely the International Association of Pancreatology (IAP; ‘Fukuoka guidelines’) [4•, 5•, 6•], American College of Gastroenterology (ACG) [7•], American Gastroenterological Association (AGA) [8•] and European Study Group on Cystic Tumours of the Pancreas [9•, 10•]. They all agree that the risk of malignancy should be weighed against life expectancy and co-morbidity. Confusingly, the recommended surveillance strategies differ between guidelines (Table 3). Incentive large-scale prospective registries of individuals undergoing cyst surveillance (e.g. PACYFIC-registry; www.pacyfic.net) are needed to accumulate unbiased data and develop evidence-based guidelines.

**Table 3:** An overview of four most recent guidelines on diagnosis and management of pancreatic cystic neoplasms [6, 8, 10, 95]

	Revised EU guideline (2018)	Revised Fukuoka guideline (2017)	ACG guideline (2018)	AGA guideline (2015)
Diagnostic work-up	MRI: 1^st^ choice CT: 2^nd^ choice* EUS: supplementary FNA: in case of mural nodules, septations or indefinite imaging Serum 19-9	MRI: 1^st^ choice CT: 2^nd^ choice* EUS: for worrisome features FNA: in case of indefinite imaging; discouraged in case of high-risk/worrisome features Serum 19-9	MRI: 1^st^ choice EUS/CT: alternative FNA: in case of indefinite imaging, high risk characteristics, cysts > 2 cm (differentiation mucinous and non-mucinous) Serum 19-9	MRI: 1^st^ choice EUS: high-risk features FNA: in case of ≥ 2 high-risk features or significant change of high-risk feature
MD-/MT-IPMN: indications for surgery	Surgically fit patients	Surgically fit and ≥ 1 high-risk stigmata (see below)	Reference to multidisciplinary group in case of main-duct involvement	Not mentioned
BD-IPMN: high-risk features/indications surgery	Absolute indications: Solid mass Enhancing mural nodule ≥ 5 mm MPD ≥ 10 mm HGD/carcinoma in cytology Jaundice Relative indications: Cyst growth ≥ 5 mm/year Cyst size ≥ 4 cm Enhancing mural nodule < 5 mm MPD 5–9.9 mm Serum CA 19-9 ≥ 37 U/ml New-onset DM Acute pancreatitis	High-risk stigmata: Enhancing mural nodule > 5 mm MPD > 10 mm Jaundice Worrisome features: Growth ≥ 5 mm/2 years Cyst size ≥ 3 cm Enhancing mural nodule < 5 mm Enhancing thickened cyst wall MPD 5–9 mm PD calibre change Elevated serum CA 19-9 Pancreatitis	High-risk characteristics: Mural nodule/solid component MPD > 5 mm PD calibre change + atrophy Cyst size ≥ 3 mm Cyst growth > 3 mm/year HGD/carcinoma in cytology Jaundice Acute pancreatitis Elevated serum CA 19-9 New-onset DM	High-risk features: Solid component Dilated MPD Cyst size ≥ 3 cm
Duration surveillance	As long as fit for surgery	As long as fit for surgery	As long as fit for surgery Individualized approach for age 76–85 years	Discontinue after 5 years if no significant change has occurred
Surveillance intervals	6 months (1^st^ year), then yearly	< 1 cm: 6 months, then 2 yearly 1–2 cm: 6 months (1^st^ year), yearly (2 years), then 2 yearly 2–3 cm: 3–6 months (1^st^ year), then yearly > 3 cm: 3–6 months	< 1 cm: 2 years 1–2 cm: 1 year 2–3 cm, clear IPMN/MCN: 6–23 months. Shorter interval for new-onset DM or cyst growth > 3 mm/year	At years 1, 3 and 5
Indication for surgery	≥ 1 Absolute indication ≥ 1 Relative indication without significant co-morbidities ≥ 2 Relative indications for patients with significant co-morbidities	≥ 1 High risk stigmata ≥ 1 Worrisome feature and ≥ 1 of the following: Definite mural nodule, MPD involvement Suspect cytology Consider: cyst > 2 cm in young and fit patient	Decided by multidisciplinary team. Referral in case of jaundice or ≥ 1 of the following: MPD > 5 mm, Cyst size ≥ 3mm Calibre change MPD MPD involvement HGD/PDAC cytology Mural nodule	Solid component and dilated MPD and/or concerning features on EUS-FNA
Surveillance after resection	Malignancy: according to PDAC guidelines HGD/MD-IPMN: 6 months (1^st^ 2 years), then yearly LGD: as non-operated	Malignancy: according to PDAC guideline 2x/year in case of one of the following: family history of PDAC, surgical margin with HGD, non-intestinal type IPMN Other patients Every 6–12 months	Malignancy: according to PDAC guidelines HGD: every 6 months Low-/intermediate grade dysplasia: every 2 years	Dysplasia/malignancy: every 2 years If not: no FU (unless MT-IPMN or family history of PDAC)

EU, European; ACG, American College of Gastroenterology; AGA, American Gastroenterological Association; MRI, magnetic resonance imaging; CT, computer tomography; EUS, endoscopic ultrasound; FNA, fine-needle aspiration; CH-EUS, contrast-enhanced harmonic EUS; CA 19-9, cancer antigen 19-9; DM, diabetes mellitus; FU, follow-up; PDAC, pancreatic ductal adenocarcinoma; LGD, low-grade dysplasia; HGD, high-grade dysplasia

*To identify calcifications, for tumour staging or for surveillance of recurrence in case of PDAC

According to all guidelines, the presence of mural nodules or solid components is most predictive for malignant disease. Mural nodules are present in 36–70% of IPMN patients with invasive disease and the size of the mural nodule is correlated with the risk of malignancy [13, 20, 31, 86]. Additionally, a thickened cyst wall is present in ~ 65% of patients with invasive disease (OR 4.80; 95% CI 1.16–14.36) [13, 87]. In case of doubt, contrast-harmonic endoscopic ultrasound (CH-EUS) helps to differentiate between mucin and a solid component by the presence of small blood vessels in the latter.

Although cyst size is associated with invasiveness, treatment should not be determined by size alone, since small cysts do not exclude invasiveness and large cysts do not always harbour malignancy [18, 19, 88–90]. The surveillance intervals in both Fukuoka and ACG guidelines are based on cyst size in the absence of a more practical surrogate [6•, 7•]. The cyst growth appears to be more predictive. A growth of > 2 mm/year is related to a 45% 5-year risk of developing malignancy versus 1.8% in slowly growing cysts [96–98]. Due to a recorded size difference between the different imaging modalities, it is recommended not to alternate modalities between follow-up visits [7•, 10•, 87, 94].

The mean MPD diameter is significantly larger in patients with malignant disease. Some guidelines use a 10-mm cut-off value, as absolute indication for surgery [6•, 10•]. This is disputable, since the risk of malignancy is already increased to 59% for patients with a pancreatic duct width between 5 and 9 mm [22]. The AGA and ACG guidelines recommend EUS-FNA in cysts associated with a dilated MPD (ACG cut-off > 5 mm, AGA non-specified) [7•, 8•, 17, 19, 22, 95].

According to the EU, Fukuoka and ACG guidelines, the duration of surveillance should be lifelong. The AGA guideline recommends stopping surveillance in the case of a stable cyst after 5 years. Interestingly, Kwong et al. (2016) [96] found an eightfold higher mortality from non-pancreatic causes than from pancreatic cancer after 5 years of surveillance in low-risk BD-IPMN. On the other hand, multiple studies detected high-risk features in asymptomatic BD-IPMN patients after a follow-up period of more than 5 years [97–99]. Additionally, Del Chiaro et al. (2017) [100] found an IPMN-related mortality of 5.8% after 10 years of follow-up in patients without high-risk features at baseline.

After resection of IPMN, lifelong surveillance is recommended, as long as the patient is able and willing to undergo surgery [6•, 7•, 8•, 10•]. He et al. (2013) [101] estimated the chance of developing a new lesion after resection of non-invasive IPMN at 1.6% after 1 year, 14% after 5 years and 18% after 10 years and the chance of invasive pancreatic cancer ~ 0% after 1 year, 7% after 5 years and 38% after 10 years. For invasive IPMN, post-resection surveillance is recommended solely based on symptoms, similar to pancreatic cancer [6•, 10•]. However, one could argue that surveillance should restart (e.g. after ~ five years) for patients with early-stage invasive IPMN, surveillance should restart after ~ 5 years of survival.

Additionally, data about the incidence of extra-pancreatic neoplasms in patients with IPMN remains controversial, since some retrospective studies show an increased risk in other cancers (e.g. colorectal and gastric cancer) [102–105]. A large study of 1340 patients by Marchegiani et al. (2015) [36] did not find a higher incidence of extra-pancreatic neoplasms in patients with IPMN. Guidelines do not recommend additional imaging (e.g. CT) for surveillance of extrapancreatic malignancies in patients with IPMN [6•, 7•, 8•, 10•].

### Treatment

Guidelines recommend that surgery should be performed by an experienced surgeon in a high-volume centre after consultation and joint decision by a multidisciplinary group with pancreatic expertise. Especially, advanced age and the presence of co-morbidity are related to postoperative mortality of non-pancreatic cause [106–108]. On the other hand, early surgery could be considered in younger patients with no co-morbidity [9•, 10•].

MD-IPMN and MT-IPMN justify a more aggressive treatment approach than BD-IPMN. In general, surgery should be offered as this is justified by the high prevalence of invasive disease (MD-IPMN 11–81%; MT 20–65%) and the high disease-specific mortality (23 per 1000 patient years; 95% CI 12–52) for untreated MD-IPMN and MT-IPMN [109].

For BD-IPMN, the guidelines are inconsistent and compared in Table 3. The Fukuoka guidelines recommend surgery in the case of ≥ 1 high-risk stigmata or ≥ 1 worrisome features and one of the following: mural nodule ≥ 5 mm, suspicious MPD, suspicious cytology [6•]. The EU guideline is similar, yet in the case of surgical indication, age and the presence of co-morbidity are advised to be taken into account [10•]. ACG stresses the need of decision-making by a multidisciplinary pancreatic group [7•].

In case of suspected malignancy, an oncological resection should be performed. For all IPMNs, intraoperative frozen section examination of the resection margins is recommended. For patients with MD-IPMN or MT-IPMN, intraoperative pancreatoscopy with frozen section of intraductal biopsies can be considered [10•]. Patients with positive margins have a worse survival and extended resection is recommended [15]. Cysts in multifocal IPMNs should be approached autonomously due to their distinct behaviour; the most suspicious lesion(s) should be removed. A total pancreatectomy is only recommended in the case of multiple worrisome features throughout the pancreas or post-surgical recurrence in the remnant pancreas and is performed in 3-37% of the patients. Severe weight loss, diarrhoea (exocrine insufficiency) and/or hypoglycaemic episodes (i.e. brittle diabetes; endocrine insufficiency) are regular consequences of total pancreatectomy [116 117]. However, the majority experiences severe weight loss, diarrhoea (exocrine insufficiency) and/or hypoglycaemic episodes in relation to brittle diabetes (endocrine insufficiency) [110, 111]. The survival rates of total pancreatectomy after 1 and 3 years are 80 and 65%, respectively [111].

Pancreaticoduodenectomy ( Whipple procedure) and distal pancreatectomy are performed in 42-70% and 13-47% of the cases [13, 15, 17, 32, 118]. These procedures are related to complications in 25% of patients, such as anastomotic leakage or stenosis, pancreatic fistula, intra-abdominal abscess, pancreatitis, pancreatic pseudocyst, cholangit is, delayed gastric emptying, ascites, diarrhoea or pneumonia [19]. In-hospital morbidity is 37%, and the in-hospital and 30-day mortality 1.4% and 2.7, respectively [15, 119].

## Prognosis

### Recurrence after surgery

The overall recurrence rate for IPMN is ~ 11–20% (median 58–73 months), which increases to 65% in the case of malignant IPMN [7•, 24, 114, 115]. For BD-IPMN, ~ 40% is multifocal, which may explain the frequent early recurrence of IPMN in the remnant pancreas (12.5%; mean follow-up 28 months) [116]. Additionally, an increased age, BMI, number of resected lesions as well as an initial location in the pancreatic tail, invasiveness and a family history of PDAC are predictors of recurrence or disease progression [117, 118]. The estimated chance to develop a new primary IPMN and related invasive pancreatic cancer after 5 years is 14 and 7%, respectively [101, 114, 119]. The recurrence rate for MD-IPMN is higher than for BD-IPMN. The dysplasia grade in the resection specimen is the most important predictor of the (severity of) recurrence [24, 114, 120].

### Survival

A large observational study by Marchegiani et al. (2015) [114] found a 5-year survival after resection of 77% for all IPMNs, 69% for MD-IPMN and 82% for BD-IPMN, with a median time to survival of 17, 13 and 24 months, respectively. Vanella et al. (2018) [109] performed a meta-analysis and found a disease-specific mortality of 23 for all IPMN, 32 for MD-IPMN and 5 for BD-IPMN per 1000 patient years.

In case of invasiveness the overall survival decreases significantly (95% vs. 49%)[114]. Low-grade dysplasia exhibits a similar survival as high-grade dysplasia. In the case of invasive disease, the survival is significantly lower. Of all patients with IPMN-associated adenocarcinoma, 53% has lymph-node metastases, 58% peri-neural and 33% vascular invasion [114, 121].
